# Supplementary material for: Multi‐dataset identification of innovative feature genes and molecular mechanisms in keratoconus
Source: J Cell Mol Med. 2024 Sep 19;28(18):e70079. doi: 10.1111/jcmm.70079 (PMC11412914; doi:10.1111/jcmm.70079)
Supplement: Supplementary file 2 — Material S2. [file JCMM-28-e70079-s002.zip › 7.GSEA analysis/1.ARL11/KEGG_NON_SMALL_CELL_LUNG_CANCER.html]

Details for gene set KEGG\_NON\_SMALL\_CELL\_LUNG\_CANCER[GSEA]

|  || Dataset | ARL11.group.cls#h\_versus\_l |
| Phenotype | group.cls#h\_versus\_l |
| Upregulated in class | h |
| GeneSet | KEGG\_NON\_SMALL\_CELL\_LUNG\_CANCER |
| Enrichment Score (ES) | 0.19088598 |
| Normalized Enrichment Score (NES) | 0.8793039 |
| Nominal p-value | 0.61165047 |
| FDR q-value | 1.0 |
| FWER p-Value | 1.0 |
Table: GSEA Results Summary

  

Fig 1: Enrichment plot: KEGG\_NON\_SMALL\_CELL\_LUNG\_CANCER      
 Profile of the Running ES Score & Positions of GeneSet Members on the Rank Ordered List

  

| SYMBOL | TITLE | RANK IN GENE LIST | RANK METRIC SCORE | RUNNING ES | CORE ENRICHMENT || 1 | RARB | na | 584 | 0.459 | 0.0838 | Yes |
| 2 | PIK3R3 | na | 2180 | 0.284 | 0.1001 | Yes |
| 3 | PIK3CG | na | 2870 | 0.247 | 0.1344 | Yes |
| 4 | PLCG1 | na | 4334 | 0.189 | 0.1337 | Yes |
| 5 | PIK3R1 | na | 6079 | 0.135 | 0.1131 | Yes |
| 6 | PLCG2 | na | 6096 | 0.135 | 0.1422 | Yes |
| 7 | SOS1 | na | 6194 | 0.132 | 0.1684 | Yes |
| 8 | CDKN2A | na | 8387 | 0.085 | 0.1238 | Yes |
| 9 | PIK3R2 | na | 8783 | 0.078 | 0.1296 | Yes |
| 10 | AKT2 | na | 9348 | 0.070 | 0.1286 | Yes |
| 11 | TP53 | na | 9447 | 0.068 | 0.1408 | Yes |
| 12 | RXRA | na | 9938 | 0.061 | 0.1400 | Yes |
| 13 | SOS2 | na | 9955 | 0.061 | 0.1528 | Yes |
| 14 | PIK3R5 | na | 10118 | 0.059 | 0.1611 | Yes |
| 15 | PRKCA | na | 10617 | 0.052 | 0.1582 | Yes |
| 16 | BRAF | na | 10628 | 0.052 | 0.1694 | Yes |
| 17 | KRAS | na | 10630 | 0.052 | 0.1808 | Yes |
| 18 | PDPK1 | na | 10673 | 0.052 | 0.1909 | Yes |
| 19 | EGFR | na | 11115 | 0.046 | 0.1883 | No |
| 20 | PIK3CA | na | 13512 | 0.023 | 0.1242 | No |
| 21 | EGF | na | 13590 | 0.022 | 0.1269 | No |
| 22 | RASSF5 | na | 15399 | 0.008 | 0.0766 | No |
| 23 | ERBB2 | na | 16056 | 0.003 | 0.0584 | No |
| 24 | RB1 | na | 16201 | 0.002 | 0.0548 | No |
| 25 | E2F2 | na | 16509 | 0.000 | 0.0459 | No |
| 26 | CCND1 | na | 16546 | -0.000 | 0.0449 | No |
| 27 | MAP2K2 | na | 16997 | -0.003 | 0.0327 | No |
| 28 | PRKCG | na | 17545 | -0.007 | 0.0184 | No |
| 29 | E2F1 | na | 17721 | -0.008 | 0.0152 | No |
| 30 | AKT1 | na | 18085 | -0.011 | 0.0071 | No |
| 31 | HRAS | na | 18435 | -0.013 | -0.0000 | No |
| 32 | FOXO3 | na | 18679 | -0.015 | -0.0038 | No |
| 33 | CDK4 | na | 18688 | -0.015 | -0.0007 | No |
| 34 | STK4 | na | 18997 | -0.017 | -0.0059 | No |
| 35 | BAD | na | 19342 | -0.019 | -0.0116 | No |
| 36 | CASP9 | na | 20358 | -0.026 | -0.0350 | No |
| 37 | FHIT | na | 20403 | -0.027 | -0.0305 | No |
| 38 | RXRB | na | 22268 | -0.043 | -0.0749 | No |
| 39 | MAPK3 | na | 22614 | -0.046 | -0.0747 | No |
| 40 | GRB2 | na | 23043 | -0.051 | -0.0759 | No |
| 41 | RAF1 | na | 23336 | -0.054 | -0.0725 | No |
| 42 | PIK3CB | na | 23818 | -0.060 | -0.0732 | No |
| 43 | RASSF1 | na | 23998 | -0.062 | -0.0647 | No |
| 44 | MAPK1 | na | 24626 | -0.071 | -0.0672 | No |
| 45 | CDK6 | na | 24642 | -0.071 | -0.0520 | No |
| 46 | E2F3 | na | 25055 | -0.077 | -0.0471 | No |
| 47 | PIK3CD | na | 25759 | -0.087 | -0.0483 | No |
| 48 | ARAF | na | 26195 | -0.093 | -0.0404 | No |
| 49 | TGFA | na | 27293 | -0.114 | -0.0469 | No |
| 50 | AKT3 | na | 27547 | -0.120 | -0.0279 | No |
| 51 | NRAS | na | 27790 | -0.126 | -0.0073 | No |
| 52 | MAP2K1 | na | 31070 | -0.223 | -0.0530 | No |
| 53 | RXRG | na | 33017 | -0.323 | -0.0382 | No |
| 54 | PRKCB | na | 33863 | -0.399 | 0.0249 | No |
Table: GSEA details [plain text format]

  

Fig 2: KEGG\_NON\_SMALL\_CELL\_LUNG\_CANCER      
 Blue-Pink O' Gram in the Space of the Analyzed GeneSet

  

Fig 3: KEGG\_NON\_SMALL\_CELL\_LUNG\_CANCER: Random ES distribution      
 Gene set null distribution of ES for **KEGG\_NON\_SMALL\_CELL\_LUNG\_CANCER**
